# A Roadmap Toward the Definition of Actionable Tumor-Specific Antigens

**DOI:** 10.3389/fimmu.2020.583287

**Published:** 2020-12-03

**Authors:** Robin Minati, Claude Perreault, Pierre Thibault

**Affiliations:** ^1^ École Normale Supérieure de Lyon, Université Claude Bernard Lyon I, Université de Lyon, Lyon, France; ^2^ Institute for Research in Immunology and Cancer, Université de Montréal, Montréal, QC, Canada; ^3^ Department of Medicine, Université de Montréal, Montréal, QC, Canada; ^4^ Department of Chemistry, Université de Montréal, Montréal, QC, Canada

**Keywords:** tumor-specific antigens, neoantigens, immunopeptidome, proteogenomics, alternative antigens, cancer immunotherapy, pan-cancer antigen research

## Abstract

The search for tumor-specific antigens (TSAs) has considerably accelerated during the past decade due to the improvement of proteogenomic detection methods. This provides new opportunities for the development of novel antitumoral immunotherapies to mount an efficient T cell response against one or multiple types of tumors. While the identification of mutated antigens originating from coding exons has provided relatively few TSA candidates, the possibility of enlarging the repertoire of targetable TSAs by looking at antigens arising from non-canonical open reading frames opens up interesting avenues for cancer immunotherapy. In this review, we outline the potential sources of TSAs and the mechanisms responsible for their expression strictly in cancer cells. In line with the heterogeneity of cancer, we propose that discrete families of TSAs may be enriched in specific cancer types.

## Introduction

The selection of “Cancer immunotherapy” by the journal *Science* as the breakthrough of the year in 2013 (1) properly illustrates how promoting the patients’ immune response against cancer cells has revolutionized the field of anticancer therapies. More recently, the Nobel prize award to Allison and Honjo, highlights the significance of immunotherapies, and how it changed the way we treat several types of cancers over the past decades. In contrast to classical treatment (i.e., surgery, chemotherapy, and radiation therapy) which target directly and aspecifically the tumoral cells, immunotherapies target the host’s immune system to initiate, augment and/or reestablish an efficient antitumoral immune response (2). Several types of immunotherapies including vaccines (3), antibodies (4), oncolytic viruses (5), immune checkpoint inhibitor (ICI) therapies (6, 7), and T cell-based immunotherapies are currently used in the clinic.

Independently from their mechanisms of action, all of these therapies rely on the ability of the patient’s adaptive immune system to discriminate between healthy (i.e., normal or stressed) cells and cancerous ones. At the molecular level, this distinction is possible because tumoral cells undergo a series of genetic and epigenetic changes leading to the generation of new self-derived antigens which are generally termed tumor-specific antigens (TSAs) or neoantigens. While neoantigens are defined as the subset of TSAs generated by genetic variations only found in the genome of a tumor, TSAs refers to all the antigen types which are specific to cancer cells (8). Because they are not expressed by the medullary thymic epithelial cells (mTECs), which are responsible for the establishment of the central tolerance (9), TSAs represent a source of potentially immunogenic neoepitopes able to be recognized and targeted by the host’s T cells (10). However, before being recognized by T cells through their T cell receptor (TCR), these antigens need to be sequentially processed and presented at the surface of the tumoral cell *via* major histocompatibility complex class I (MHC I) molecules. For MHC I, the antigen processing starts in the cytosol where intrinsic proteins—originating from the self in normal cells or altered-self in tumoral cells—are cleaved into peptides by the proteasome and some aminopeptidases (11). Then, the generated peptides are translocated in the endoplasmic reticulum (ER) *via* transporter associated with antigen processing (TAP) and further processed by the ER aminopeptidase 1 and 2 to reach a size ranging between 8 and 10 residues (11). Peptides are then loaded into the peptide cleft of a MHC I molecule and if the MHC I–peptide complex is stable enough, it is exported at the cell surface and referred to as MHC I–associated peptide (MAP) (11).

MAPs have a central role in T cell activation and more generally in anti-tumoral immunity. However, the question of how we can efficiently identify cancer-specific MAPs and, more generally TSAs, is now rising. As mentioned above, tremendous progress has been made in the way we treat many tumors. Unfortunately, the development of new antitumoral immunotherapies is now partially limited by the difficulty to identify targetable TSA and more precisely cancer-specific MAPs that could be used to initiate an efficient antitumoral immune response. Indeed, immunotherapeutic strategies which are used in the clinic either (i) bypass MHC I presentation (e.g., chimeric antigen receptor, CAR-T cell therapy) or (ii) skip the step of cancer-specific MAPs identification because they rely on a pre-existing antitumoral T cell response (e.g., ICI therapies).

**Figure 1:**
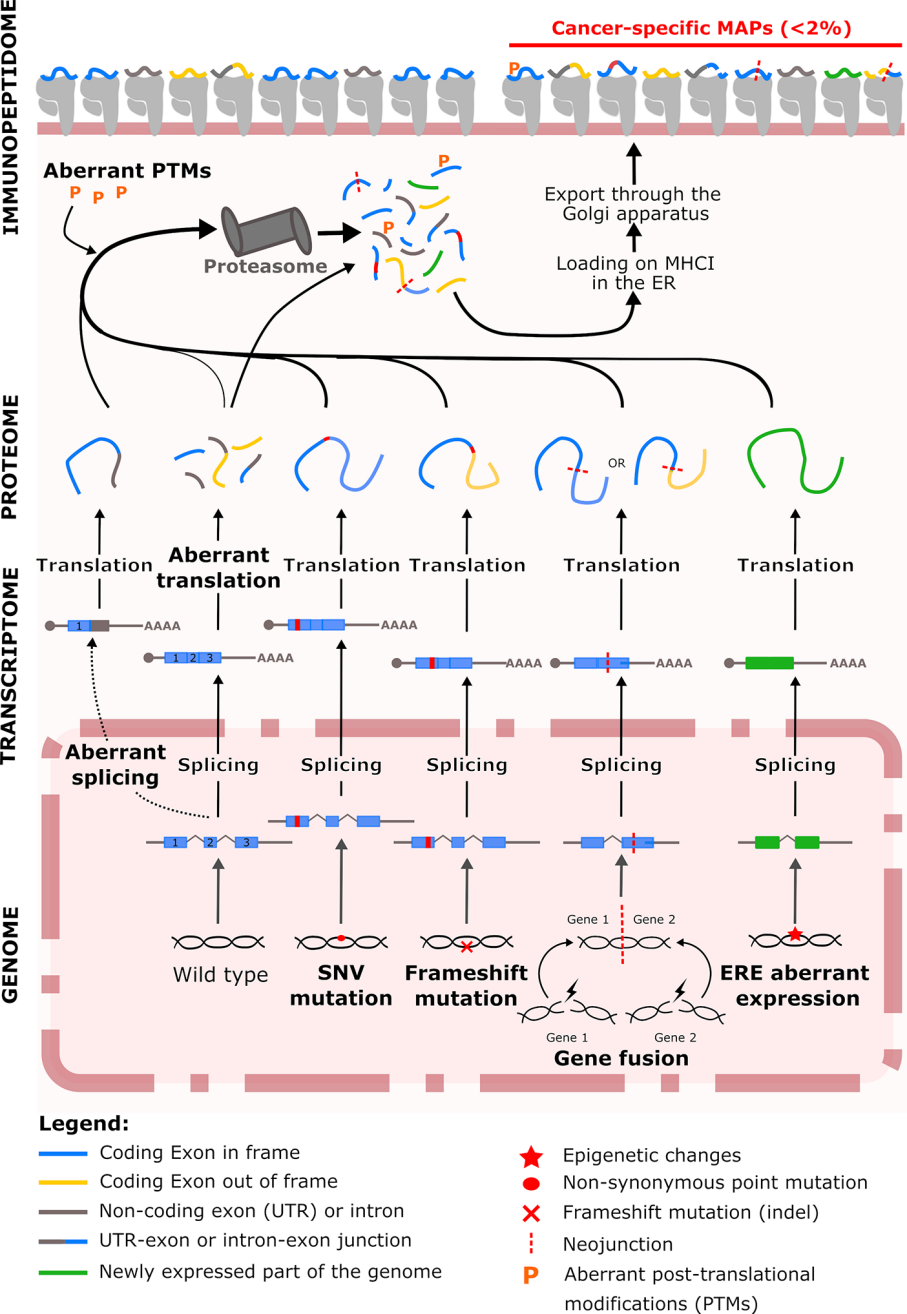
Overview of the tumor-specific antigen production in tumor cells. At the genomic level, cancer cells accumulate tumor-specific genetic and epigenetic changes. Within genomic alteration, single-nucleotide variants (SNVs) represent an historic source of immunogenic neoantigens. Insertions/deletions (indels) or gene fusion events increase the tumor immunogenicity by generating peptide deriving from the out-of-frame translation of coding exons. Epigenetic alterations induce the aberrant expression of endogenous retroelements (EREs) which generated non-mutated cancer-specific peptides with a high immunogenic potential. In addition to genomic alterations, Post-translational modifications (PTMs) cancer-specific events such aberrant splicing events, ribosomal translation and PTMs also contribute to the generation of cancer-specific major histocompatibility complex class I (MHC I)-associated peptides (MAPs). ER: endoplasmic reticulum.

In this review, we highlight the different sources of TSAs and the mechanisms responsible for their production in cancer cells with the objective to facilitate the identification of multiple targetable cancer-specific MAPs within tumors (**Figure 1**). Although tumor-associated antigens such as cancer-testis antigens can represent valuable source of antigens, we do not discuss them here and focus our topic to antigens absent from healthy tissue. In line with the heterogeneity of cancer, we also propose that discrete families of TSAs may be enriched in specific cancer types.

## Single Nucleotide Variant Neoantigens: A Relatively Meager Source of Tumor-Specific Antigens for Immunotherapies

Single nucleotide variants (SNVs)—and by extension double nucleotide variants—are commonly referred to as non-synonymous point mutations and can result, like most DNA damages, from (i) DNA replication errors, (ii) DNA enzymatic modification, (iii) exogenous or endogenous mutagen exposures, or (iv) defective DNA repair.

SNVs are the most common genetic variation (12) and they have been considered for a long time as the most promising source of TSAs driving antitumoral responses. This mostly explains why the vast majority of the studies aiming to identify “tumoral neoantigens” have concentrated their efforts in detecting these non-synonymous point mutations located in known exons. Based on the original hypothesis that the TSA number of a given tumor is proportional to its SNV burden (commonly referred to as mutation burden), SNV-derived MAPs appeared as particularly good immunotherapeutic candidates for the most mutated tumor types—i.e., metastatic melanoma and lung cancers (13). However, all studies based on whole-exome or ribonucleic acid sequencing (WES and RNAseq respectively), combined or not with mass spectrometry (MS) analysis, could only identify a very limited number of SNV-derived MAPs. For example, in native human melanoma, which represents the cancer type having the highest mutation burden (13), Bassani-Sternberg et al. could only identify 11 cancer-specific immunopeptides (14). While this might be a limitation of MS sensitivity, it is puzzling that in these ICI-responding tumors, further investigations have shown that most of the identified cancer-specific SNVs were not immunogenic (14). The fact that most predicted TSAs are not validated by MS can be explained by two factors. Some of these TSAs are probably false negatives caused by the sensitivity of shotgun MS analyses (15). However, in-depth genomic analyses suggest that most false negatives are true negatives. Indeed, no evidence was found supporting the negative selection (via immunoediting) of SNV-containing predicted TSAs (16, 17). Furthermore, response to ICI in patients with lung cancer did not correlate more with SNV-containing predicted TSAs than with the global mutation burden (18). These data suggest that the number of genuine SNV-containing TSAs has been overestimated in many studies. Tran et al. estimated that the *in vivo* immunogenicity—i.e., the capacity for antigens to be recognized by a tumor infiltrating lymphocyte (TIL)—of the whole exonic non-synonymous mutations of human gastrointestinal cancers was ranging between 0.009% to 1.25% (19). Although in some cases few neoantigens are sufficient to control the tumor or reach a therapeutic effect (20, 21), these results suggest that most cancer-specific MAPs able to trigger an antitumoral T cell response are likely not originating from exonic point mutations, and that other sources of neoantigens should be explored to drive future antitumoral immunotherapies (22).

## Mutational-Frameshift Neoantigens: A Promising Source of Tumor-Specific Antigens for Renal Cell Carcinoma, Homologous Recombination-Deficient Tumors, and High Microsatellite Instability (MSI-H) Tumors

After SNVs, nucleotide insertions or deletions (indels) represent the second most abundant type of mutation in cancer (23). With a size that ranges between one and more than 60 base pairs (bp), single and trinucleotides indels are the most common events and represent 68% and 13% of all indel events, respectively (23). Biologically and by extension immunologically, all indels are not equals and we can distinguish two types: (i) a minority of in-frame indels that lead to the production of lowly immunogenic shorter or longer variants and (ii) a majority of frameshift indels that give rise to truncated protein variant containing new (potentially highly immunogenic) fragments derived from the out-of-frame translation of a coding exon (23). Since frameshift indels (i) have the potential to generate more neoantigens than SNVs (24) and (ii) differ greatly from the germline sequences used for the establishment of the central tolerance, earlier reports suggested that they might serve as better immunotherapeutic targets (24, 25). In accordance with this proposal, several studies have shown a positive correlation between the indel burden and the presence of TILs inside the tumor or the response of the patients to ICI therapies (23, 24, 26–29). More importantly, in a context of non-relevant nonsense-mediated mRNA decay (NMD), truncated mutant proteins resulting from frameshift mutations have been reported to be extensively degraded by the proteasome system (30, 31). While this mechanism protects tumor cells from the potentially harmful effects of truncated proteins, it also promotes the generation of frameshift-derived peptides and their presentation at the cell surface (30).

Interestingly, indel burden also varies significantly across malignancies, though differences were noted regarding their exact proportions in various cancer types. Niavarani et al. reported that the indel proportion across cancers globally ranges between 1.3% and 29.1% (23) while this proportion was estimated between 1% and 12% by Turajlic et al. (24). It is noteworthy that in both studies, renal cell carcinomas, RCCs (i.e., chromophobe renal cell carcinoma, renal papillary cell carcinoma and renal clear cell carcinoma, KIRC) are classified among the cancers with the highest proportion of indels. At the therapeutic level, this is particularly promising because RCCs contain relatively few SNVs and the high proportion of indel opens new perspectives for neoantigen discovery. The presence of frameshift indel-derived antigens could explain the infiltration of RCCs by TILs and their good response to ICI therapies (32). In support of this, Hansen et al. recently reported that TILs from six patients with RCC could recognized both SNV- and frameshift-derived neoantigens (33). While frameshift-derived neoantigens represented only about 16% of the predicted TSAs, they corresponded to 21% of the immunogenic MAPs identified in the study.

In addition to being particularly abundant in RCC, indels have also been reported to accumulate importantly in both homologous-recombination (HR)– and DNA mismatch repair (MMR)–impaired tumors (13). Impaired HR repair pathway has been observed in subpopulations of breast, ovarian and pancreatic cancer where it is associated with an accumulation of numerous large deletions (up to 50 bp) along the genome (13). HR is normally used by dividing cells to guide the error-free repair of double-strand breaks (34) but when it is not available, other error-prone mechanisms ensure the breakpoint junction (13, 35, 36). In ovarian cancer, HR-deficiency is associated with a favorable clinical prognostic (36, 37). This is most likely due to an increase of both the tumoral neoantigen load and immunogenicity resulting from frameshifting indels accumulation. In support of this hypothesis, Strickland et al. showed that for high grade serous ovarian cancer, HR-deficient tumors presented more TILs, higher expression levels of programmed death 1 (PD-1) and programmed cell death ligand 1 (PD-L1) and more putative neoantigen than HR-proficient ones (36). Similar results were reported for triple-negative breast cancer (38) but, to our knowledge, such studies were not yet conducted in pancreatic cancer. Although the exact contribution of the indel frameshift-derived neoantigens to the immunopeptidome of HR-deficient tumor cells still needs to be determined, this class of antigen seems to play an important role in the antitumoral T cell response and represent a promising immunotherapeutic target for subsets of ovarian, breast and pancreatic cancer.

Normally involved in the correction of indel loops and bp mismatches occurring during DNA replication, the MMR pathway is crucial to maintaining the microsatellite stability across the genome (39). As a result, its impairment in tumors leads to what is referred to as a microsatellite instability (MSI). Both MMR-deficiency and high MSI (MSI-H) have been primarily documented in both familial (Lynch syndrome) and sporadic subsets of colorectal cancers with a quite high prevalence (40–42). They are now reported in a wide range of malignancies including ovarian (43), endometrial (44), gastric (45), and prostate (46) cancers. On a pan-cancer scale, the endometrial, colon and gastric cancers are the cancer types displaying the three highest proportions of MSI-H cases (47). On an immunologic level, the high accumulation of somatic mutations by MSI-H tumors suggests that they should display several neoantigens (48). In support to this hypothesis, Le et al. demonstrated that pembrolizumab—an anti-PD-1 antibody—was effective in a wide range of MMR-deficient solid tumors (49, 50), opening the way for the first FDA tissue-agnostic approval of an ICI therapy. At the same time, they also showed that the response to pembrolizumab was associated with the *in vivo* expansion of T cell clones specific for tumoral indel-derived neoantigens providing a proof-of-concept of the relevance of targeting frameshift-derived neoantigens in MSI-H malignancies.

## Gene Fusion Neoantigens Are Rather Rare but Recurrent Across Malignancies

Gene fusion events are less frequent than SNVs and indel mutations, and consist in the juxtaposition of two previously independent coding sequences by (i) structural rearrangements at the genomic level (i.e., chromosomal translocation, inversion or deletion), (ii) transcription read-through of adjacent genes (51, 52), or (iii) *trans*- and *cis*-splicing of pre-mRNAs (53–55). In these three cases, the result is the production of a fusion transcript that can be translated into what we refer to as a fusion or chimeric protein. Although fusion events also occur in non-tumoral cells (56, 57), many cancer-specific fusion proteins have already been associated with a different malignancies including leukemia (58), sarcoma (59), breast (60), bladder (61), colon (62), and lung (63) cancers where they can be used as diagnosis and prognostic markers.

So far, most of the studies conducted on oncogenic fusion proteins have been focusing on either leukemia—i.e., acute myeloid leukemia, acute lymphocytic leukemia, or chronic myeloid leukemia—or sarcoma (64) where SNV burdens is relatively low (13, 65). In these malignancies, the hope of developing a vaccine was stimulated by the observation that some gene fusion events, such as the translocations t(11;22)(p13;q12) and t(12;22)(q13;q12), are particularly recurrent in given specific cancer subtypes (66). In a pan-cancer analysis of fusion events, Vellichirammal et al. have recently shown that the 40 most recurrent fusion events cover a wide spectrum of malignancies (67), and as such represent a promising source of multivalent neoantigens that could be used to drive cross-cancer immunotherapies.

Moreover, several gene fusions have been reported to act as driver mutations favoring tumorigenesis (68). Mechanistically, this oncogenic influence can be exerted either by altering the expression or activity of tumor suppressor or proto-oncogenes or by forming a fusion product with oncogenic properties (e.g., a constitutively activated tyrosine kinase domain). Because, in this case, fusion products are a source of oncogenesis and they are functionally linked with tumor fitness, targeting them may be more beneficial clinically than targeting other types of passenger mutations. Several pharmaceutical inhibition-based therapies targeting oncogenic fusion products have already shown promising results in the clinics (69–71). Regarding immunotherapies, several attempts were made to design fusion neoantigen-based vaccines but could only demonstrate moderate clinical efficacy (72–75). In these studies, even though the fusion peptides used for vaccination were able to activate the patients’ T cells, all immunizations were performed with a single gene fusion epitope that may have favored the emergence of vaccine-resistant sub-clones. Although driver mutations have been shown to be highly clonal during the early stages of cancer, they tend to become highly heterogeneous and sub-clonal at later stages of the disease (76). Therefore, a driving fusion protein that may be essential for the survival of a transformed cell during cancer initiation can be completely absent from part of its progeny once the tumor is well established. In addition to this loss of clonality, cancer cells also develop different mechanisms enabling them to escape the immune surveillance. These mechanisms include the expression of immune checkpoints (7), a complete (77), or partial (78) loss of MHC I expression and the epigenetic silencing of neoantigens recognized by the immune system (79). These observations suggest that immunotherapies against fusion products-derived neoantigens would be more effective against early-stage cancers rather than later ones. On a pan-cancer level, patients with malignancies characterized by relatively low SNV and indel burden and a minimal immune-infiltration, such as leukemia (8), sarcoma (8), adenoid cystic carcinomas (80), or head and neck tumors (80), will most likely benefit the most from fusion product targeting. In the context of vaccine development, fusion proteins are a meaningful source of neoantigens, and their therapeutic value could be enhanced by combining several “driver” and “passenger” neoepitopes originating from different fusion proteins, and by including ICI in the vaccination protocol to minimize the risk of immune evasion (80).

## Endogenous Retroelements-Derived Tumor-Specific Antigens: A Predominant Source of Non-Mutated Antigens for a Vaccine Against Cancer

Endogenous retroelements (EREs) represent about 42% of the human genome (81) and result from the integration of transposable elements into our genome millions of years ago. They comprise both long terminal repeat (LTR) elements (i.e., human endogenous retroviruses, HERVs, and mammalian apparent LTR-retrotransposons)—and non-LTR elements—which include long and short interspersed nuclear elements (LINEs and SINEs, respectively). Following their long co-evolution with human, the vast majority of EREs are now truncated and/or mutated and have lost their capacity to transpose in the genome (82–84). For those still able to “replicate” a strict epigenetic repression is maintained on their open reading frames (ORFs) to prevent the insertional mutagenesis and chromosomal rearrangements associated with their expression (85). Considered for a long time as “junk” DNA, the remnants of retroelements still contains functional promoters, enhancers, ORFs, splice donor/acceptor sites and polyadenylation sites able to impact cell physiology (85) and can contribute to several key processes of our development and adulthood (86–88). In line with this, Larouche et al. recently reported that various levels of ERE transcripts can be found in all human somatic tissues and that their expression is particularly predominant in mTECs which are responsible for T cell negative selection (89). These findings suggest that some antigens derived from these “domesticated” EREs are tolerated by the immune system.

In the context of cancer, the alteration of the epigenetic landscape or the use of demethylating therapies can result in the loss of repression marks along the genome and dysregulate ERE expression leading to the transcription and translation of aberrantly expressed EREs (aeEREs) (90–94). These aeERE have been reported to affect cancer progression through both pro- and antitumoral mechanisms (95). Previous reports indicated that aeERE could generate viral-like neoantigens able to increase both the antigenicity and immunogenicity of tumor cells (89, 94). Unlike the ERE-derived antigens expressed in normal tissues, those restricted to cancer cells (i.e., aeEREs) can be recognized by the immune system although they originate from non-mutated sequences. Indeed, several aeERE-derived MAPs were shown to activate CD8^+^ T cells in both B-lymphoblastoid cell line and KIRC (89, 96). Because aeEREs can produce non-mutated immunogenic neoantigens, they are now considered as a particularly attractive source of TSAs for the development of cancer vaccines. Unlike mutated neoantigens which are “private”, non-mutated TSA, such as aeEREs, are very likely to be shared across tumors and malignancies.

On a pan-cancer level, Attig et al. compared the expression levels of “cancer-specific LTR element-overlapping transcripts” (CLTs) across 31 cancer types and showed that the three malignancies with the highest number of CLTs were respectively the testicular germ cell tumors (TGCTs), the esophageal carcinoma and the ovarian serous cystadenocarcinoma (82). Although most of the overlap in CLT expression was observed in related tissues such as KIRC and renal papillary cell carcinoma, the study highlighted that 44 CLTs were shared by ten or more cancer types (82). Although a pan-cancer study including LINEs and SINEs is still needed, this LTR analysis supports the notion that aeEREs represent meaningful targets for the generation of shared TSAs.

## The Post-Transcriptional Antigens and Their Rising Interest for Immunotherapies

Although most studies have focused on TSA classes arising from genomic alterations (e.g., SNV-, indels-, gene fusion-, and ERE-derived antigens), other classes of TSA exist and can still contribute to the development of antitumoral immunotherapies. Broadly referred to as post-transcriptional TSAs, this wide class of antigen regroups antigens derived from aberrant (i) alternative splicing, (ii) ribosomal events, and (iii) post-translational modifications (PTMs).

### Aberrant Splicing-Derived Tumor-Specific Antigens

Alternative splicing of premature messenger RNAs (pre-mRNAs) is responsible for the diversification of both the transcriptome and the proteome of eukaryotic cells. This cellular process explains how one protein-coding gene can generate multiple alternative transcripts, also called variants, and give rise to different protein isoforms which are structurally and sometimes functionally different (97). Tightly regulated in time and space in normal cells, alternative splicing is carried out by the spliceosome machinery, and plays a key role in both cellular differentiation and identity (98). On a mechanistic level, alternative splicing events traditionally include intron retention, exon skipping, the use of alternative 5’- or 3’-splice site which lead to the retention of exon fragments, and exon mutual exclusion. However, since alternative promoters and alternative polyadenylation sites can generate transcripts with alternative 5’- and 3’-ends, they are sometimes considered as alternative splicing events although they are not directly carried out by the spliceosome machinery (99).

In cancer, it is now well established that both aberrant alternative splicing events (i.e., novel transcripts absent in normal cell) and alterations in the ratio of alternatively spliced transcripts occurs in a wide range of malignancies including breast (100, 101), brain (102), colon (103), prostate (103, 104), lung (105), and ovarian (101) cancers. Although both are cancer landmarks, only aberrant splicing events can generate cancer-specific transcript that can be translated into new protein isoforms and produce immunogenic TSAs. Arising either from *cis*-acting splice junction mutations (106) or *trans*-acting spliceosome dysregulation (107, 108), aberrantly spliced transcripts lead to the formation and translation of cancer-specific junctions termed neojunctions. Based on the position and the nature of the neojunction—i.e., (i) in-frame exon-exon junctions (ii) out-of-frame exon-exon junctions, (iii) exon-intron junctions, or (iv) exon-untranslated region (UTR) junctions, the impact on protein’s function and immunogenicity can be significant. At the functional level, all aspects of tumor development, progression, and response to treatments can be affected by aberrant alternative splicing and several known aberrantly splice variants have been shown to affect key processes such as metabolism, apoptosis, cell cycle control, angiogenesis, invasiveness, metastatic potential, and resistance to treatments of tumors (99). Similar to gene fusions, cancer-specific variants deriving from aberrant alternative splicing events can be functionally linked to the fitness of the tumor. However, unlike gene fusion products that can only be turned on or off, these splicing variants offer additional levels of plasticity which seem to be used by tumors to evade both immune surveillance (109) and therapies. In line with that, several aberrant spliced variants appear to be implicated with the resistance to several antitumoral treatments such as imatinib (110, 111), poly adenosine triphosphate (ADP)-ribose polymerase (PARP) inhibitor (112), cisplatin (112, 113), and tamoxifen (114). Although this link between aberrant protein isoform and drug resistance still needs to be confirmed, recent reports suggest that targeting aberrant splicing could resensitize cancer cells to existing therapies (99).

While mutations responsible for the occurrence of an aberrant splicing event were initially presumed to occur only in the consensus intronic dinucleotide splice donor (GT) or acceptor (AG) sites, it is now clear that other mutations can also affect RNA maturation (115–118). As a result, many mutations originally misannotated as silent, missense, insertion/deletion or nonsense mutations are now being considered as *cis*- and *trans*-acting splicing mutations (106). Unlike *cis*-acting splicing mutations which only affect the local splicing of the genes carrying them, *trans*-acting splicing mutations affect directly or indirectly the regulation of the spliceosome machinery and therefore the splicing process of many genes. By looking for splicing quantitative trait loci (sQTLs), Kahles et al. recently identify seven *trans*-sQTLs, two of which were associated with mutations in genes encoding the core spliceosome factors splicing factor 3b subunit 1 (SF3B1) and U2 small nuclear RNA auxiliary factor 1 (U2AF1) (107). Unexpectedly, other identified mutated genes (i.e., transcriptional adaptor 1, TADA1, protein phosphatase 2 scaffold subunit A alpha, PPP2R1A, epidermal growth factor receptor, EGFR, and isocitrate dehydrogenase 1, IDH1) were not previously known to impact the splicing of other genes. While the mechanistic basis behind this association still needs to be clarified, the landscape of *trans*-acting splicing variant continues to evolve. In another TCGA study, Seiler et al. identified far more genetic alterations able to impact other genes’ alternative splicing in various ways by focusing on mutation affecting 119 known splicing factor genes (108). While it may still be too early to determine how much *trans*-acting splicing mutations contribute to the immunopeptidome of a tumor, this class of mutation has the potential to generate much more neojunctions than its *cis*-acting homologue.

At the scale of different cancer types, like for SNVs or indels, the “neojunction/aberrant splicing burden” of the different malignancies varies considerably (107). On average, ovarian serous cystadenocarcinoma, liver hepatocellular carcinoma, esophageal carcinoma, and stomach adenocarcinoma are the four cancer types displaying the highest numbers of neojunctions. However, when looking at the median values, the global ranking changes drastically suggesting that important internal variations do exist within cancer types. In that context, in addition to this cancer-based analysis, it seems that a cancer-subtype approach could be used to identify more patient subgroups likely to benefit from the development of aberrant splicing targeting therapies.

Despite the fact that it is still unknown if the increase of aberrant splicing events in tumors is positively selected or not, splicing-derived cancer-specific proteins can potentially be used to inform cancer immunotherapies. In their studies, both Khales et al. and Jayasinghe et al. predicted that aberrantly splicing events were much more likely to generate TSAs able to bind MHC I then SNV mutations (106, 107). Although the translation of several of the alternative splicing-derived putative neoepitopes could be validated using publicly available MS data from other studies, most of their prediction relied on both RNAseq data and prediction algorithms. Since aberrant splicing is predominantly associated with low-abundance isoforms (119), the presentation of the corresponding peptides and their immunogenic potentials require experimental validation before they can be of immunotherapeutic value.

### Tumor-Specific Antigen Derived From Aberrant Translation

Aberrant ribosomal translation events (ARTEs), sometimes called non-canonical translation events, correspond to the translation of either allegedly non-coding sequences or coding sequences in a non-canonical reading frame. Such events generally include non-canonical initiation, elongation and termination events. Briefly, a non-canonical initiation event occurs when the ribosome does not start the translation at the primary AUG codon—but at a non-primary AUG codon (120) or at a near-cognate start codon (CUG, UUG, or GUG) (121)—as a result of a start codon scan-through (122), a translation reinitiation (123) or the presence of an internal ribosome entry site (IRES) on the messenger RNA (124). Non-canonical elongation events happen when a frameshift occurs spontaneously during elongation and lead to the translation of a part of the protein in a non-canonical reading frame. Some slippage-prone sequences present within transcripts have already been reported to promote what is called a programmed ribosomal frameshift (125). Non-canonical termination events, although rare, are possible and consist of either a stop-codon read-through (126)—some stop codons such as UGA and UAG appears to be leakier than UAA—or a ribosomal frameshift at the stop codon. When such ARTEs occur, they lead to the production of non-canonical proteins and cryptic MAPs.

While cryptic MAPs were initially observed as marginal and irrelevant, this view is now changing as we get a better understanding of their immunotherapeutic potentials. Recent studies indicated that at least 10% of the MAP repertoire is represented by cryptic peptides that are common among individuals carrying the same MHC I alleles (123). Interestingly, cryptic MAPs are involved in the establishment of the central tolerance and the priming of CD8^+^ T cells in mice (127). They are also recognized and targeted by both TILs (128–131) and auto-reactive T cells (120) in human. As their relevance to adaptative immunity becomes clearer, TSA research is virtually expanded from the ~2% of protein-coding genes to the ~75% of the transcribed genome (132). As such, ARTEs are redefining translation events at the whole transcriptome level while aberrant splicing events discussed in the previous section are delineating the boundaries of exons and introns.

ARTEs are found in both normal cells and tumoral cells, though their products differ depending on the genetic and epigenetic instability associated with cancer cells. This difference leads to the generation of cancer-specific cryptic MAPs that are relevant targets for vaccine development (93, 133, 134). Indeed, MAPs deriving from aberrant expression of non-mutated non-coding regions of the genome are much more likely to be shared by multiple tumors than randomly mutated sequences. Moreover, contrary to canonical MAPs, the generation of cryptic MAPs can be enhanced by inflammatory stimuli (135) such as type I interferon or tumor necrosis factor alpha (TNFα) and by drugs (136) such as aminoglycoside that might be used to increase the global immunogenicity of cold tumors.

Since cryptic MAPs cannot be identified using canonical protein databases, Laumont et al. have recently developed a proteogenomic-based approach to identify both of canonical and cryptic MAPs specific to tumor cells (e.g., mutated and aberrantly expressed TSA) (93). In parallel of this, two proof-of-principle studies established that MAPs can also be identified using reference databases built from ribosome profiling (Ribo-seq) (137, 138). Ribo-Seq is based on the sequencing of mRNA fragments protected for ribonuclease digestion by their location within the ribosome decoding site. It provides quantitative information on the nature of translated mRNAs including their reading frame and start and termination codons. While both of these approaches open new avenues for identification of cryptic MAPs which are potentially shared between patients, one unanswered question is the identification of TSAs among cryptic MAPs identified using databases built on Ribo-Seq. Indeed, this would require Ribo-Seq data for all types of normal cells.

### Tumor-Specific Antigen Derived From Post-Translation Modifications

There are approximately 300 PTMs that have been described to modify proteins in normal condition (139). Among them, we find very diverse modifications—such as acetylation, ubiquitination, glycosylation, SUMOylation, etc.—which are important to control the stability, localization, and conformation of proteins within the cell. Particularly important for the cell signaling, many PTMs have been shown to be dysregulated in the context of cancer (140–143).

Among the most studied PTMs, phosphorylation is the one associated with the largest number of diseases (144). In cancer, Reimand et al. showed, that SNV mutations affecting the phosphorylation sites could be found in nearly 90% of the tumors where they are were associated with gains or losses of signaling contributing to what they called the “oncogenic rewriting of the kinase network” (145). On an antigenic level, phosphorylated proteins were shown to be processed normally by the antigen presentation pathway of both normal and tumoral cells (146–152). In line with the idea that they could be used for immunotherapies, several studies reported that phosphorylated peptides, but not their dephosphorylated counterparts, could activate T cells in the mice model (150, 153). Although their immunogenic potential has not been demonstrated in human, attempts are currently being made to integrate peptide phosphorylation into MHC I binding prediction tools (154). If they succeed, prediction tools should facilitate the detection of phosphorylated peptide and clarify their potential as a source of TSAs.

In addition to phosphorylation, other PTMs such as citrullination, ubiquitination and O-glycosylation might also contribute to both the antigenicity and immunogenicity of cancer cells in different ways. Citrullination results from the deimination of arginine residues into citrulline by a peptidyl-arginine deiminase (PAD). Despite being involved in several physiological processes, citrullination is predominantly known for its involvement in several autoimmune disorders including rheumatoid arthritis, multiple sclerosis, and type I diabetes where it was shown to be immunogenic (155–159). It is important to note that this PTM has also been identified in cancer (160). Citrullination levels seems to be higher in ovary, uterus, colon, bladder, breast, liver, lung, esophagus, kidney, and prostate tumors than in their corresponding normal tissues due to the overexpression of either PAD4 or PAD2 (161–163). Although the presentation of citrullinated MAPs on MHC I molecules has never been demonstrated, citrullination was shown to increase peptides binding affinity for HLA‐DRB1 (a MHC class II allele) (164, 165) which could then be recognized by both mice and human repertoires of “cytotoxic” CD4^+^ T cells (166–168).

The β O-linked N-acetylglucosamine (O-GlcNAc) is a ubiquitous PTM modifying both serine and threonine residues and is involved in cell signaling of in all eukaryotic cells (169). This modification is reversibly attached and removed from its substrates in the cytosol or the nucleus of the cell by the O‐linked N‐acetylglucosaminyltransferase (OGT) and the β‐N‐acetylglucosaminidase (OGA), respectively (169). In normal cells, O-GlcNAcylation modulates several important biological functions such as the enzymatic and transcription activities, protein turnover, protein-protein interactions, and subcellular localization of several proteins (170, 171). Dysregulations of the O-GlcNAcylation as well as aberrant expression of OGT and/or OGA have been observed in cancer where they are associated with increased cancer cell proliferation and survival, invasiveness, and metastasis (171). Because O-GlcNAcylated proteins are present at the level of the cytosol, O-GlcNAcylated MAPs were shown to be displayed at the cell surface and activate T cells (172, 173). The resolution of two different MHC I–glycopeptide structures by X-ray crystallography highlighted that the accessibility of the O-GlcNAc group to the TCR was key for the T cell reactivity (174). More recently, using MS, Malaker et al. could identified 36 unique O-GlcNAcylated MAPs from primary human leukemic and Epstein-Barr virus-transformed B cell (175). While the MAPs they identified presented various levels of glycosylation and methylation, five out of the seven tested could activate T cells from healthy donors. Although these antigens have not been proven tumor-specific, the authors reported that 92% of the identified O-GlcNAcs MAPs could not be detected in their healthy tissue samples (175).

While phosphorylation, citrullination or O-GlcNAcylation can be explored as potential sources of immunogenic TSAs, other PTMs such as ubiquitination do not directly provide tumor-specific epitopes but have been shown to affect peptide presentation. Ubiquitin is usually used as a degradation signal in the cell: when a protein reaches a threshold of ubiquitination, it is addressed to the proteasome where it is hydrolyzed into peptides (176). These peptides (among others) are a source of endogenous antigens for the MHC I presentation pathway. As a result, due to its key role in providing peptides for the MHC I immunosurveillance, dysregulation of the ubiquitination in cancer could lead to a modification of the immunopeptidome landscape. In case of a decrease in ubiquitination (intrinsic or pharmacologically-induced (177)), more peptides from the ubiquitin-independent presentation pathway will be presented at the cell surface. This includes peptides originating from small defective ribosome products (DRiPs) and aberrant translation products which seems to generate MAPs in a proteasome-independent manner (11). While the potential of PTMs for immunotherapies is still not yet fully accessed, preliminary results are promising and open additional perspective to target cancer.

## From Identified Tumor-Specific Antigens to Actionable Therapeutics: Validation and Selection

By definition the identification of a candidate requires to determine the correct amino acid sequence as well as the precise nucleic sequence its originates from. When the TSA candidate sequence is closed from similar to the reference sequence like it is the case for SNVs, the identification is relatively easy especially as soon as WES or RNAseq data are available. However for other TSA classes, it can be more challenging. In the case of frameshift mutations, indels are difficult to identify from Sanger and next-generation sequencing which is why special tools such as pVACseq (178), Neopepsee (179), MuPeXI (180), Epidisco (181) and Antigen garnish (182) have been developed. While pVACtools, Epidisco, and Antigen.garnish also support the prediction of gene fusion-derived peptides, other tools such as INTEGRATE-neo have been specifically designed to predict fusion neoantigens (183). Because no prediction software is currently able to deal with repetitive regions, the prediction of ERE-derived TSA can only be made combining quantifiers such as hervQuant (184)or RepeatMasker (185) with a classic epitope prediction software such as NetMHCpan (186).

To face issues associated with the large search space of unbiased identification, one may want to conduct targeted searches with databases from human mRNA annotated sequences with associated variation information derived from the Single Nucleotide Polymorphism Database (dbSNP) and remove all non-polymorphic information (187). Searches for alternative reading frames, transcripts from non-coding regions (93) or EREs (89) can be achieved separately and results combined subsequently. As mentioned in this review, TSA classes have different abundances in different tumor types. Therefore, knowing which class might be predominant in a tumor of interest, could be used to guide identification by reducing search space.

Once identified, the actionability of the TSA is determined based on five parameters which evaluate/validate different aspects of its therapeutic potential.

First, TSA candidates have to be validated as “truly” tumor-specific. Because targeting antigens which are also expressed in healthy tissues could result in severe side effects or autoimmunity, Laumont et al. developed a stringent validation strategy based on the resources gathered by the GTEx consortium (93). In their study, candidates were only considered as “true” TSA if their corresponding reads were absent from the transcriptomes of a wide range healthy tissue. Although well adapted for most TSA classes, this validation strategy could not be apply to TSAs deriving from aberrant translations or PTMs. For these particular classes, their absence from healthy tissues could only be validated at the proteomic level, but, to our knowledge, it has never been done.

The second key criteria to assess TSA’s actionability is their immunogenic potential. To be targetable, a presented TSA has to be recognized by a TCR and able to trigger T cell activation. This is usually determined *ex vivo* by interferon gamma ELISpot, but a wide range of well-established assays have been described and can quantify other aspects of the T cell-dependent immunogenicity than interferon gamma production (188). We can also mention that many efforts are currently being made to develop machine-learning approaches to predict the immunogenicity of a given peptide from its sequence (189). Although this could both faster and ease the selection of therapeutic epitopes, this approach is still limited by the type of data available to train the programs.

Third, it is also important to estimate the incidence/prevalence of newly discovered TSA on a pancancer, tumoral and subtumoral scale. The more an immunogenic antigen is shared between patients (i.e., frequent within a cancer type/subtype or across malignancies), the higher its therapeutic interest will be. This evaluation is usually performed by looking for TSA-corresponding transcripts in a large number of cancer transcriptomes from the TCGA database. By using this approach, Zhao et al. recently showed that 78% and 18% of the transcripts encoding for aberrantly expressed TSA in ovarian cancer were respectively expressed by at least 10% and 80% of the ovarian cancer samples (190). While this parameter is key to establishing the wide scale potential of an antigen, here again this strategy could not be applied for TSAs deriving from aberrant translations or PTMs.

Forth, the large-scale therapeutic potential of a candidate peptide is also affected by its MHC restriction (i.e., the number and frequencies of MHC I alleles it can bind to). If an immunogenic TSA is shared by an extensive number of tumors but only presented by a rare MHC I allele, its therapeutic interest for universal therapies will be decreased compared with another antigen which can be presented by a large portion of the population. TSA binding profiles are generally determined using MHC I–binding prediction tools such as NetMHC or NetMHCpan (186, 191) but needs to be experimentally validated either using a proteogenomic approach but ideally this should be done by T2 or RMA-S peptide binding assay (192, 193).

Fifth, the potential of TSAs will finally depend on the type of immunotherapy/strategy which is considered. In the particular context of vaccine design, all classes of TSA are not necessarily suitable for all vaccination strategy (i.e., DNA/mRNA-based vaccination (194), peptide-based vaccination (195), or TSA-loaded antigen presenting cell infusion (196, 197)). For example, PTM-derived TSA are not suitable for the development of DNA/mRNA-based vaccines. Similarly, private antigens would be less suitable for the development of a broad universal vaccine than shared non-mutated TSAs.

## Conclusion

While the vast majority of the studies aiming to identify tumor antigens have concentrated their efforts in the detection of SNVs with limited therapeutic results, the possibility of enlarging the repertoire of targetable TSAs by looking at alternative classes of antigens opens new perspectives for the development of cancer immunotherapies. In line with recent improvements in both MAPs detection and prediction methods, our knowledge of these “alternative” sources of TSAs has remarkably increased over the past few years. From the maintenance of the genetic and epigenetic information at the genomic level to the ribosomal translation and PTM, every step of protein expression is susceptible to be dysregulated in cancer. While dysregulation may lead to the generation of specific types of TSA with their own features, they do not occur uniformly across malignancies. Therefore, more than “how”, the true question is now choosing “what” to identify. What class of neoantigen is the most likely to be predominant in this given type/subtype of tumor? What class of antigen is the most suitable for immunotherapy?

Given the fast-evolving nature of tumors and their genetic heterogeneity, it is very likely that future immunotherapies will need to target more than one TSA at once. While some neoantigens are derived from source proteins essential for tumor fitness, most TSAs that are now identified using conventional proteogenomic approaches are not necessarily required for tumor survival. To be efficient, immunotherapies must target multiple TSAs from different origins to cover the diversity of tumor subclones and prevent drug resistance.

## Author Contributions

All authors contributed to the design of the review. RM conducted literature search and wrote the first draft of the manuscript. All authors contributed to the article and approved the submitted version.

## Funding

This study was supported by grants from the Canadian Cancer Society (#705604) and the Leukemia and Lymphoma Society and Canada. RM is supported by a scholarship from MITACS. IRIC proteomics facility is a Genomics Technology platform funded in part by the Canadian Government through Genome Canada.

## Conflict of Interest

The authors declare that the research was conducted in the absence of any commercial or financial relationships that could be construed as a potential conflict of interest.

The reviewer AA declared a past co-authorship with two of the authors CP and PT to the handling editor.
